# Non-linear relationship between pulse pressure and the risk of prediabetes: a 5-year cohort study in Chinese adults

**DOI:** 10.1038/s41598-024-52136-x

**Published:** 2024-02-15

**Authors:** Zhenhua Huang, Fangxi Wang, Xiaoyong Xiao, Dehong Liu, Zhe Deng

**Affiliations:** Department of Emergency, Shenzhen Second People’s Hospital, Shenzhen, 518037 Guangdong China

**Keywords:** Biomarkers, Endocrinology

## Abstract

Previous research has established a strong link between pulse pressure (PP) and diabetes, but there is limited investigation into the connection between PP and prediabetes. This study aims to explore the potential association between PP and prediabetes. A retrospective cohort study encompassed 202,320 Chinese adults who underwent health check-ups between 2010 and 2016. Prediabetes was defined in accordance with the World Health Organization criteria, indicating impaired fasting glucose, with fasting blood glucose levels ranging from 6.1 to 6.9 mmol/L. To assess the PP-prediabetes relationship, we employed Cox regression analysis, sensitivity analysis, and subgroup analysis. Cox proportional hazards regression, coupled with cubic spline functions and smooth curve fitting, helped elucidate the non-linear PP-prediabetes relationship. Upon adjusting for confounding factors, we observed a positive association between PP and prediabetes (HR 1.15, 95% CI 1.11–1.18, *P* < 0.0001). Participants in the fourth quartile (PP ≥ 51 mmHg) had a 73% higher likelihood of developing prediabetes compared to those in the first quartile (PP < 36 mmHg) (HR 1.73, 95% CI 1.52–1.97, *P* < 0.0001). Moreover, the relationship between PP and prediabetes was non-linear. A two-piece Cox proportional hazards regression model identified an inflection point at 40 mmHg for PP (P for log-likelihood ratio test = 0.047). Sensitivity and subgroup analyses corroborated the robustness of our findings. Our study reveals a non-linear correlation between PP and prediabetes, signifying an increased risk of prediabetes when PP levels exceed 40 mmHg. This discovery has significant clinical implications for early prediabetes prevention and intervention, ultimately contributing to improved patient outcomes and quality of life.

## Introduction

Prediabetes is a condition characterized by abnormal blood glucose levels that do not meet the diagnostic criteria for diabetes^1^. According to the World Health Organization (WHO) criteria, prediabetes encompasses impaired glucose tolerance (IGT), with 2-h blood glucose levels between 7.8 and 11.0 mmol/L during an oral glucose tolerance test, or impaired fasting glucose (IFG), with fasting blood glucose levels ranging from 6.1 to 6.9 mmol/L^2^. Prediabetes significantly increases the risk of progressing to diabetes, with an estimated 50–70% of individuals developing diabetes within 5 years^2,3^. According to the 9th edition of the International Diabetes Federation (IDF) Diabetes Atlas 2019, approximately 7.5% (374 million people) of adults aged 20–79 worldwide have prediabetes, with the highest prevalence in North America and the Caribbean. In 2021, Mary R. Rooney et al. conducted an analysis of over 90 high-quality studies from 43 countries, estimating a global prevalence of 9.1% (464 million people) for impaired glucose tolerance, projected to increase to 10.0% (638 million people) by 2045. Additionally, the global prevalence of impaired fasting glucose in 2021 was 5.8% (298 million people), projected to increase to 6.5% (414 million people) by 2045^2^. Based on WHO definitions of IFG and IGT, the burden of prediabetes is substantial and continues to rise worldwide. Therefore, effective monitoring of prediabetes is essential for implementing diabetes prevention policies and interventions.

Blood pressure is a crucial indicator for assessing and managing the risk of cardiovascular diseases, as it reflects the state of the circulation. It plays a pivotal role in evaluating an individual’s cardiovascular disease risk, monitoring treatment effectiveness, guiding treatment strategies, and aiding in early cardiovascular disease detection^4,5^. Elevated blood pressure is a significant risk factor for diabetes development^6,7^. This may be attributed to hypertension causing insulin resistance and affecting insulin secretion and insulin receptor sensitivity^8,9^. Traditionally, studies have primarily utilized systolic and diastolic blood pressure to assess cardiovascular risk. However, pulse pressure (PP), defined as the difference between systolic and diastolic blood pressure, is also a valuable indicator of blood pressure and is frequently used to measure arterial elasticity and assess the cardiovascular system’s functional status^10^. A low PP may indicate reduced arterial elasticity or impaired cardiac pumping function, resulting in inadequate organ and tissue perfusion^11^. Prolonged elevated PP may increase the strain on the heart and blood vessels, thereby increasing the risk of cardiovascular disease^11,12^. Some studies have found higher PP may be associated with an increased risk of cardiovascular diseases such as arteriosclerosis, coronary heart disease, and stroke^13–16^. Several studies have found that higher PP is associated with an increased risk of diabetes^17,18^. This suggests that PP could serve as a novel marker for predicting the onset of diabetes.

However, research on the relationship between PP and prediabetes is currently limited. A study from 2018 investigated the connection between PP and insulin resistance in individuals with prediabetes. The findings revealed a positive correlation between PP and insulin resistance, suggesting that an increase in PP could independently predict insulin resistance^8^. Nevertheless, these observations are based on observational studies and cannot establish a causal relationship between PP and prediabetes. Consequently, further research is necessary to validate and gain a better understanding of the association between PP and prediabetes.

To delve deeper into the link between PP and prediabetes, we have designed a large-scale cohort study involving 202,320 participants from 32 locations in 11 cities across China. Our goal is to analyze the potential association between PP and the future risk of prediabetes. By doing so, we aim to demonstrate PP as a promising marker for predicting prediabetes, offering valuable insights for prediabetes screening and diabetes prevention strategies.

## Results

### Baseline characteristics of participants

Table 1 shows the demographic and clinical characteristics of the study participants. In the present study, 202,320 individuals were included. The mean age was 41.57 ± 12.36 years old. 109,410 (54.08%) individuals were men, and 92,910 (45.92%) individuals were women. A total of 202,320 individuals developed prediabetes after a follow-up period of an average of 3.12 years. PP divided into four groups based on quartiles: Q1 ≤ 36 mmHg; 37 < Q2 ≤ 42 mmHg; 43 < Q3 ≤ 50 mmHg; Q4 > 51 mmHg. As shown in Table 1, compared to Q1, the Q4 group had higher levels of age, systolic blood pressure (SBP), diastolic blood pressure (DBP), BMI, AST, ALT, TG, LDL-C, TC, BUN, Scr, FPG1 and FPG2. Additionally, the Q4 group had a higher proportion of males, smokers, drinkers, and individuals with a family history of diabetes. In comparison to Q4, the Q1 group had higher levels of HDL-C.

**Table 1:** The baseline characteristics of participants.

PP (quartile) (mmHg)	Q1 (≤ 36)	Q2 (37–42)	Q3 (43–50)	Q4 (≥ 51)	*P* value
participants	49,325	45,002	54,047	53,946	
Age (years)	40.16 ± 10.13	40.33 ± 10.49	40.70 ± 11.54	44.78 ± 15.49	< 0.001
Height (cm)	165.54 ± 7.99	166.10 ± 8.15	166.99 ± 8.27	166.92 ± 8.74	< 0.001
Weight (kg)	61.54 ± 11.55	63.24 ± 11.75	65.14 ± 11.93	67.06 ± 12.37	< 0.001
BMI (kg/m^2^)	22.34 ± 3.15	22.81 ± 3.17	23.25 ± 3.22	23.96 ± 3.38	< 0.001
SBP (mmHg)	105.24 ± 10.87	112.31 ± 10.65	119.93 ± 10.80	134.38 ± 14.21	< 0.001
DBP (mmHg)	73.92 ± 10.77	72.75 ± 10.49	73.61 ± 10.46	75.09 ± 10.90	< 0.001
TC (mmol/L)	4.63 ± 0.86	4.65 ± 0.87	4.68 ± 0.89	4.78 ± 0.94	< 0.001
TG (mmol/L)	1.20 ± 0.91	1.25 ± 0.93	1.32 ± 0.99	1.43 ± 1.06	< 0.001
HDL-c (mmol/L)	1.39 ± 0.31	1.38 ± 0.31	1.37 ± 0.30	1.36 ± 0.30	< 0.001
LDL-c (mmol/L)	2.72 ± 0.66	2.73 ± 0.67	2.76 ± 0.67	2.83 ± 0.70	< 0.001
ALT (U/L)	16 (11.8, 24.1)	17 (12.0, 26.0)	18.2 (13.0–28.0)	19.7 (14.0–29.2)	< 0.001
AST (U/L)	21 (18.0, 25.0)	21.2 (18.0, 26.0)	22 (18.5, 26.4)	23 (19.0, 27.8)	< 0.001
BUN (mmol/L)	4.52 ± 1.15	4.57 ± 1.15	4.65 ± 1.17	4.79 ± 1.22	< 0.001
Scr (μmol/L)	67.50 ± 14.98	68.78 ± 15.18	70.63 ± 15.16	72.43 ± 17.12	< 0.001
FPG1 (mmol/L)	4.76 ± 0.56	4.81 ± 0.55	4.86 ± 0.54	4.97 ± 0.52	< 0.001
FPG2 (mmol/L)	5.00 ± 0.47	5.02 ± 0.48	5.05 ± 0.48	5.15 ± 0.51	< 0.001
Sex					< 0.001
Male	21,633 (43.84%)	22,180 (49.29%)	31,329 (57.97%)	34,278 (63.54%)	< 0.001
Female	27,712 (56.16%)	22,822 (50.71%)	22,718 (42.03%)	19,668 (36.46%)	
Smoking status					< 0.001
Current smoker	2581 (18.38%)	2414 (19.32%)	3113 (20.16%)	3025 (19.95%)	
Ever smoker	496 (3.53%)	484 (3.87%)	727 (4.71%)	685 (4.52%)	
Never	10,964 (78.09%)	9598 (76.81%)	11,603 (75.13%)	11,452 (75.53%)	
Drinking status					< 0.001
Current drinker	216 (1.54%)	290 (2.32%)	332 (2.15%)	373 (2.46%)	
Ever drinker	1770 (12.61%)	1788 (14.31%)	2501 (16.20%)	2394 (15.79%)	
Never	12,055 (85.86%)	10,418 (83.37%)	12,610 (81.66%)	12,395 (81.75%)	
Family history of diabetes					< 0.001
No	48,164 (97.61%)	44,052 (97.89%)	52,986 (98.04%)	53,104 (98.44%)	
Yes	1181 (2.39%)	950 (2.11%)	1061 (1.96%)	843 (1.56%)	

Continuous variables were summarized as mean (SD) or medians (quartile interval); categorical variables were displayed as percentage (%).

*BMI* body mass index, *SBP* systolic blood pressure, *DBP* diastolic blood pressure, *TC* total cholesterol, *TG* triglyceride, *HDL-c* high-density lipoprotein cholesterol, *LDL-c* low-density lipoprotein cholesterol, *AST* aspartate aminotransferase, *ALT* alanine aminotransferase, *BUN* blood urea nitrogen, *Scr* serum creatinine, *FPG1* fasting plasma glucose at baseline, *FPG2* fasting plasma glucose at follow-up.

### The incidence rate of prediabetes

**Figure 1:**
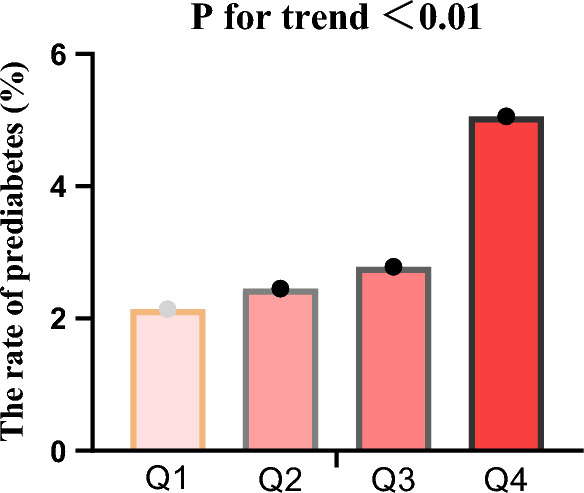
The incidence rate of prediabetes. The incidence rate of prediabetes was significantly higher in participants from Q4 compared to those in the lower PP groups (*P* < 0.01 for trend).

**Figure 2:**
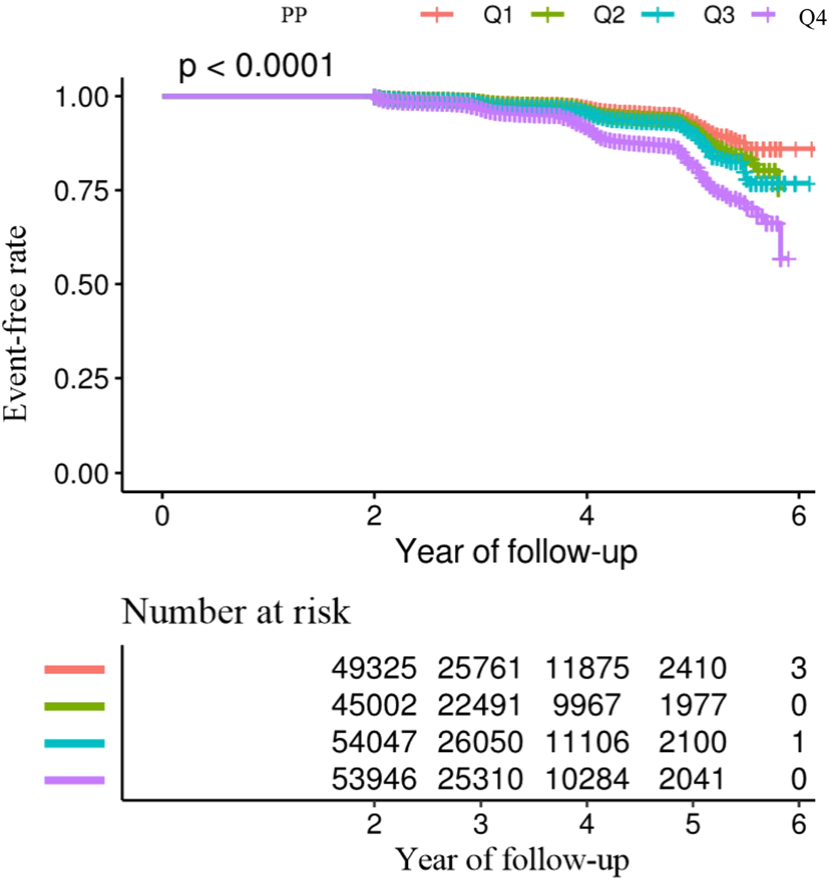
Kaplan–Meier event-free survival curve. Kaplan–Meier analysis of incident prediabetes based on PP quartiles (log-rank, *P* < 0.0001).

Among the study population, a total of 6392 individuals developed prediabetes, with an incidence rate of 3.16% (95% CI 3.08, 3.24). The cumulative reversal rates in each quartile (Q1–Q4) were as follows: Q1: 2.14%, Q2: 2.45%, Q3: 2.78% and Q4: 5.06% (Fig. 1). Compared to individuals in the lower Q1 quartile, those in the Q4 quartile had a significantly lower reversal rate (*P* < 0.001, trend test) (Fig. 2). The incidence rates for Q1–Q4 were as follows: 2.14% (2.01–2.27), 2.45% (2.31–2.60), 2.78% (2.64–2.92), and 5.06% (4.88–5.25) (Table 2). Additionally, the overall incidence rate of prediabetes was 62.462 per 10,000 person-years. The incidence rates for Q1–Q4 were 67.204, 78.141, 89.628, and 164.530 per 10,000 person-years, respectively.

**Table 2:** The Incidence rate of prediabetes (% or Per 10,000 person-year).

PP (quartile)	Participants (n)	Prediabetes events (n)	Cumulative incidence (95%CI) (%)	Per 10,000 person-year
Total	202,320	6392	3.16 (3.08–3.24)	62.462
Q1	49,325	1057	2.14 (2.01–2.27)	67.204
Q2	45,002	1104	2.45 (2.31–2.60)	78.141
Q3	54,047	1501	2.78 (2.64–2.92)	89.628
Q4	53,946	2730	5.06 (4.88–5.25)	164.530
P for trend				< 0.001

As depicted in Fig. 2, the Kaplan–Meier curves demonstrate the survival probability of not progressing to prediabetes. There is a significant variation in the risk of developing prediabetes among the four PP groups (*P* < 0.0001). With an increase in PP levels, the probability of not developing prediabetes gradually declines. This indicates that the group with the highest PP exhibits the highest risk of progressing to prediabetes.

### Univariate analysis

As shown in Table 3, age, BMI, TC, TG, AST, ALT, LDL-C, BUN, Scr and PP were positively associated with the risk of prediabetes. Conversely, HDL-C showed a negative association with the risk of prediabetes. Furthermore, women exhibited a lower risk of developing prediabetes compared to men. Additionally, individuals who abstained from alcohol and tobacco had a reduced risk of developing prediabetes.

**Table 3:** Risk of prediabetes analyzed by univariate Cox proportional hazards regression.

Variable	Characteristics	HR (95% CI)	*P* value
Age (years)	41.57 ± 12.36	1.05 (1.05, 1.06)	< 0.0001
Sex			
Male	109,411 (54.08%)	Ref	< 0.0001
Female	92,910 (45.92%)	0.60 (0.57, 0.63)	< 0.0001
BMI (kg/m^2^)	23.12 ± 3.29	1.19 (1.18, 1.20)	< 0.0001
TC (mmol/L)	4.69 ± 0.89	1.38 (1.34, 1.41)	< 0.0001
TG (mmol/L)	1.31 ± 0.98	1.24 (1.23, 1.26)	< 0.0001
HDL-c (mmol/L)	1.38 ± 0.31	0.81 (0.74, 0.90)	< 0.0001
LDL-c (mmol/L)	2.76 ± 0.68	1.39 (1.33, 1.45)	< 0.0001
ALT (U/L)	23.56 ± 21.82	1.00 (1.00, 1.00)	< 0.0001
AST (U/L)	23.89 ± 12.32	1.01 (1.00, 1.01)	< 0.0001
BUN (mmol/L)	4.64 ± 1.18	1.23 (1.21, 1.26)	< 0.0001
Scr (mmol/L)	69.93 ± 15.78	1.01 (1.01, 1.01)	< 0.0001
Smoking status			
Current smoker	11,132 (5.50%)	1.0	
Ever smoker	2392 (1.18%)	0.66 (0.52, 0.83)	0.0005
Never	43,615 (21.56%)	0.56 (0.50, 0.62)	< 0.0001
Drinking status			
Current drinker	1211 (0.60%)	1.0	
Ever drinker	8453 (4.18%)	0.47 (0.36, 0.60)	< 0.0001
Never	47,475 (23.47%)	0.43 (0.34, 0.55)	< 0.0001
Family history of diabetes			
No	198,287 (98.01%)	1.0	
Yes	4034 (1.99%)	1.03 (0.88, 1.21)	0.6852
PP (mmHg)	44.62 ± 11.48	1.04 (1.03, 1.04)	< 0.0001

### The relationship between PP and prediabetes

As shown in Table 4, in the unadjusted model, the HR (95% CI) for the association between PP and prediabetes was 1.42 (1.39, 1.44). In the minimally-adjusted model, after adjusting for gender and age, the HR (95% CI) was 1.19 (1.16, 1.21). In the fully-adjusted model, after further adjusting for gender, age, BMI, TG, LDL-C, HDL-C, AST, ALT, BUN, Scr, family history of diabetes, drinking status, and smoking status, the HR (95% CI) was 1.15 (1.11, 1.18). This indicates that for every 10-mmHg increase in PP, the risk of prediabetes increases by 15%. Additionally, when we categorized PP into four groups, in the fully-adjusted model, the risk of developing prediabetes in Q4 was 1.64 times higher than in Q1 (HR (95% CI) 1.64 (1.44, 1.87).

**Table 4:** Relationship between PP and risk of prediabetes in different models.

Exposure	Crude model (HR, 95% CI) P	Model I (HR, 95% CI) P	Model II (HR, 95% CI) P	Model III (HR, 95% CI) P
PP (10 mmHg)	1.42 (1.39, 1.44) < 0.0001	1.19 (1.16, 1.21) < 0.0001	1.15 (1.11, 1.18) < 0.0001	1.19 (1.15, 1.23) < 0.0001
PP (quartile)				
Q1	Ref	Ref	Ref	Ref
Q2	1.21 (1.11, 1.32) < 0.0001	1.17 (1.08, 1.27) 0.0002	1.02 (0.88, 1.19) 0.7549	1.03 (0.89, 1.20) 0.6699
Q3	1.44 (1.33, 1.56) < 0.0001	1.32 (1.22, 1.42) < 0.0001	1.15 (1.00, 1.32) 0.0576	1.17 (1.01, 1.35) 0.0315
Q4	2.70 (2.52, 2.90) < 0.0001	1.85 (1.72, 1.99) < 0.0001	1.64 (1.44, 1.87) < 0.0001	1.73 (1.52, 1.97) < 0.0001
P for trend	< 0.0001	< 0.0001	< 0.0001	< 0.0001

Crude model: we did not adjust other covariates.

Model I: we adjusted age, sex.

Model II: we adjusted age, sex, BMI, ALT, AST, BUN, Scr, TG, LDL-c, HDL-c, family history of diabetes, drinking status, and smoking status.

Model III: we adjusted age (smooth), sex, BMI (smooth), Scr (smooth), TG (smooth), ALT (smooth), AST (smooth), LDL-c (smooth), HDL-c (smooth), smoking status, drinking status, family history of diabetes. HR, Hazard ratios; CI, confidence, Ref, reference.

### The results of sensitivity analysis

We conducted a series of sensitivity analyses to ensure the reliability of our research findings (Tables 4 and 5). Firstly, we used a Generalised Additive Model (GAM) to incorporate a continuous covariate as a curve into the equation. The results from Model III, as shown in Table 4, were consistent with those from the fully adjusted model (HR 95% CI 1.19 (1.15–1.23), *P* < 0.001). Additionally, we performed sensitivity analyses on participants with a BMI below 28. After adjusting for potential confounding variables (including age, sex, BMI, HDL-c, TG, LDL-c, BUN, Scr, ALT, AST, family history of diabetes, smoking and drinking status), the results indicated a positive association between PP and the risk of prediabetes (HR 95% CI 1.19 (1.15–1.23), *P* < 0.001). We also excluded participants aged 60 years or older for further sensitivity analysis. After adjusting for confounding variables, the results still showed a positive correlation between PP and the incidence of prediabetes (HR 95% CI 1.28(1.23–1.34), *P* < 0.001). Moreover, when excluding participants without a family history of diabetes and adjusting for relevant variables, the results demonstrated a positive association between PP and the risk of prediabetes (HR 95% CI 1.15 (1.11–1.18), *P* < 0.001) (Table 5).

**Table 5:** Relationship between PP and the risk of prediabetes in different sensitivity analyses.

Exposure	Crude model I (HR, 95% CI) P	Model II (HR, 95% CI) P	Model III(HR, 95% CI) P
PP (10 mmHg)	1.19 (1.15, 1.23) < 0.0001	1.28 (1.23, 1.34) < 0.0001	1.15 (1.11, 1.18) < 0.0001
PP (quartile)			
Q1	Ref	Ref	Ref
Q2	1.10 (0.94, 1.30) 0.2440	0.98 (0.83, 1.17) 0.0002	1.02 (0.88, 1.19) 0.7549
Q3	1.22(1.05, 1.43) 0.0101	1.12 (0.95, 1.31) 0.1725	1.15 (1.00, 1.32) 0.0583
Q4	1.94 (1.69, 2.24) < 0.0001	1.86 (1.61, 2.16) < 0.0001	1.64 (1.44, 1.87) < 0.0001
P for trend	< 0.0001	< 0.0001	< 0.0001

Crude model I was a sensitivity analysis performed after excluding participants with BMI ≥ 28 mmol/L (N = 15,967). we adjusted age, sex, ALT, AST, BUN, Scr, TG, LDL-c, HDL-c, family history of diabetes, drinking status, and smoking status.

Model II was a sensitivity analysis performed after excluding participants with age ≥ 60 mmol/L (N = 21,599). we adjusted sex, BMI, ALT, AST, BUN, Scr, TG, LDL-c, HDL-c, family history of diabetes, drinking status, and smoking status.

Model III was a sensitivity analysis performed on participants without family of diabetes. We adjusted age, sex, BMI, ALT, AST, BUN, Scr, TG, LDL-c, HDL-c, smoking status and drinking status. HR, Hazard ratios; CI, confidence, Ref, reference.

### The non-linear relationship between PP and prediabetes

**Figure 3:**
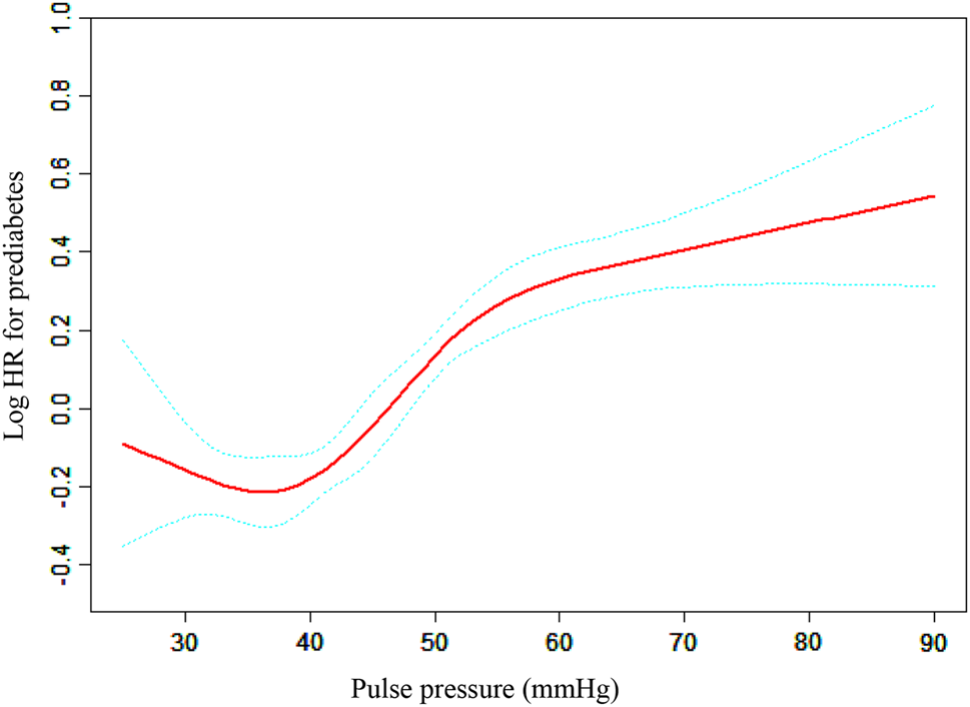
The non-linear relationship between PP and risk of predibetas. A non-linear relationship between them was detected after adjusting for gender, age, BMI, TG, HDL-c, LDL-C, AST, ALT, Scr, BUN, family history of diabetes, drinking status, smoking status.

We utilized a Cox proportional hazards regression model with cubic spline functions and found a non-linear correlation between PP and the probability of developing prediabetes (Fig. 3). To better fit the data, we employed a standard binary two-piecewise Cox proportional hazards regression model and selected the best model using the log-likelihood ratio test (Table 6). The *P* value for the log-likelihood ratio test was less than 0.05. Using a recursive technique, we identified 40 mmHg as the inflection point for PP. After the inflection point, the hazard ratio (HR) for PP and the risk of developing prediabetes was 1.17 (95% CI 1.13, 1.22, *P* < 0.0001). However, before the inflection point, the HR for PP and the risk of developing prediabetes was 1.01 (95% CI 0.89, 1.15, *P* = 0.8916), which was not statistically significant.

**Table 6:** The result of the two-piecewise Cox proportional hazards regression model.

Outcome: prediabetes	HR, 95%CI P	Value
Fitting model by standard Cox regression	1.15 (1.11, 1.18)	< 0.0001
Fitting model by two-piecewise Cox regression		
Inflection points of PP (10 mmHg)	40	40
< 40 mmHg	1.01 (0.89, 1.15)	0.8916
≥ 40 mmHg	1.17 (1.13, 1.22)	< 0.0001
P for log-likelihood ratio test		0.047

### Subgroup analysis

We conducted subgroup analysis to investigate potential additional risk factors that could influence the relationship between PP and prediabetes risk. We examined the impact of BMI, age, gender, smoking status, drinking status, and family history of diabetes as stratification factors. However, our analysis revealed that drinking status, smoking status, family history of diabetes had no significant impact on the association between PP and prediabetes risk. Additionally, there was a stronger connection between PP and risk of prediabetes in individual with age < 60 years, BMI < 24, and females (Table 7).

**Table 7:** Stratified associations between PP and risk of prediabetes by age, sex, smoking status, and drinking status.

Variable		HR (95% CI)	*P* value
Age(years)			
< 60	180,722	1.27 (1.21, 1.33)	< 0.0001
≥ 60	21,599	1.17 (1.12, 1.22)	< 0.0001
BMI			
< 24	125,936	1.20 (1.14, 1.26)	< 0.0001
24–28	60,418	1.17 (1.09, 1.20)	< 0.0001
≥ 28	15,967	1.06 (0.98, 1.14)	0.1738
Sex			
Male	109,411	1.11 (1.07, 1.16)	< 0.0001
Female	92,910	1.19 (1.13, 1.25)	< 0.0001
Drinking status			
Current drinker	1211	1.17 (0.72, 1.89)	0.5209
Ever drinker	8452	1.05 (0.86, 1.28)	0.6598
Never	47,475	1.10 (0.99, 1.21)	0.0726
Smoking status			
Current smoker	11,132	1.05 (0.89, 1.24)	0.5346
Ever smoker	2392	1.32 (0.85, 2.05)	0.2197
Never	43,615	1.08 (0.97, 1.20)	0.1804
Family history of diabetes			
Yes	198,287	1.15 (1.11, 1.18)	< 0.0001
No	4034	1.26 (0.96, 1.64)	0.0912

## Discussion

After conducting a comprehensive analysis, we discovered a non-linear relationship between PP and the risk of prediabetes. Furthermore, we pinpointed a critical threshold of 40 mmHg for PP. When PP exceeded 40 mmHg, a significant positive association with prediabetes risk was observed (HR: 1.17, 95% CI 1.13–1.22, *P* < 0.0001). However, when PP was below 40 mmHg, this association did not reach significance (HR: 1.01, 95% CI 1.08–1.15, *P* = 0.8916). Notably, a stronger connection between PP and prediabetes risk was evident in individuals under the age of 60, those with a BMI under 24, and females. These findings offer valuable insights into the relationship between PP and prediabetes risk, emphasizing the importance of monitoring PP levels when assessing prediabetes risk. Further research is warranted to uncover the underlying mechanisms and explore potential interventions tailored to individuals at risk of prediabetes based on their PP levels.

In recent years, researchers have conducted extensive studies on the relationship between PP and diabetes. Some studies have found that high PP is associated with an increased risk of diabetes. Several large-scale studies conducted in China found that an increase in PP is associated with an increased risk of type 2 diabetes^17,18^. Insulin resistance is one of the main pathophysiological mechanisms of diabetes. A study in Korea confirmed a positive correlation between PP and insulin resistance in non-diabetic adults^8^, suggesting that high PP may be associated with the development of insulin resistance.

Prediabetes is closely related to the development of diabetes, as it is considered a pre-diabetic state^19^. However, research on the relationship between PP and prediabetes is scarce. Going back to 1992, Cederholm et al. found a correlation between higher PP and impaired glucose tolerance (IGT) in a study^20^. However, this study only included 695 middle-aged subjects and only analyzed the higher PP group’s increased likelihood of developing prediabetes. The association between PP and prediabetes was not thoroughly examined. Roengrit et al. observed significantly higher PP in the impaired fasting glucose (IFG) group compared to the normal fasting glucose (NFG) group (*P* < 0.05) and confirmed a positive correlation between PP and fasting blood glucose (r = 0.20, *P* = 0.01)^21^. However, this study was a small case–control study and cannot establish a causal relationship between PP and prediabetes. To date, there is no research analyzing the causal relationship between PP and prediabetes. Based on these circumstances, we hypothesized that there might be a positive correlation between PP and the risk of developing prediabetes. To test this hypothesis, we included 202,320 participants without diabetes from 32 regions in 11 cities in China and followed them for 5 years to analyze the relationship between PP and the risk of developing prediabetes through multivariable Cox regression analysis. Our study showed a non-linear relationship between PP and prediabetes and we calculated the inflection point of PP to be 40 mmHg. When PP levels were below 40 mmHg, there was no association with the occurrence of prediabetes (HR: 1.01, 95% CI 0.89–1.15, *P* = 0.8916). However, when PP levels were above 40 mmHg, there was a 17% increased risk of developing prediabetes for every 10 mmHg increase (HR: 1.17, 95% CI 1.13–1.22, *P* < 0.0001). This suggests that we can predict the risk of developing prediabetes based on PP values.

Compared to other studies, our research delves more profoundly into the association between PP and prediabetes risk. Firstly, prior research primarily relied on cross-sectional designs, whereas our study employs a cohort study design, enhancing our understanding of the PP-prediabetes relationship among Chinese adults. Secondly, we conducted a comprehensive examination of this relationship using multivariable Cox regression analysis, accounting for variables like BMI, Scr, smoking, alcohol use, and family history of diabetes, all of which are associated with prediabetes risk^22,23^.

Our study utilized a Cox proportional hazards regression model along with cubic spline functions and smooth curve fitting to uncover the non-linear PP-prediabetes relationship. We bolstered the reliability of our findings through a series of sensitivity and subgroup analyses, affirming the stability of the PP-prediabetes relationship. Notably, we identified a stronger positive correlation in females and individuals under the age of 60, with a BMI under 24 kg/m^2^. Significantly, our study calculated the inflection point for PP, offering valuable clinical guidance for mitigating prediabetes risk.

Our study’s clinical implications are noteworthy. Firstly, individual PP values can serve as predictive indicators for prediabetes risk, enabling healthcare professionals and patients to anticipate and implement timely interventions, such as lifestyle adjustments, increased physical activity, and dietary control. Secondly, our research establishes reference values for target PP levels. When PP surpasses 40 mmHg, healthcare professionals can recommend proactive measures to mitigate prediabetes risk, including regular blood pressure monitoring, medication adjustments, and lifestyle improvements. Lastly, our findings present a novel perspective on the PP-prediabetes relationship, moving beyond linear associations commonly studied. This non-linear relationship discovery opens new avenues for exploring the mechanisms connecting PP and prediabetes. In summary, our study highlights a non-linear connection between PP and prediabetes, emphasizing increased risk when PP exceeds 40 mmHg. This discovery holds clinical significance for early prevention and intervention in prediabetes, enhancing patient outcomes and quality of life.

The mechanism by which high PP increases the risk of prediabetes is not fully understood and may be related to several factors. Firstly, high blood pressure can lead to insulin resistance, which is a reduced response of the body to insulin^24^. And high PP may be associated with increased levels of inflammation and oxidative stress^25,26^. Inflammation and oxidative stress are important mechanisms in the development of prediabetes. Finally, high PP may be related to dysregulation of the neuroendocrine system.

However, it is important to acknowledge the potential limitations of this study. Firstly, as a retrospective analysis of a cohort study, there may be unaccounted factors that could influence the relationship between PP and prediabetes, such as dietary habits and physical activity levels, despite adjusting for various factors. Secondly, the diagnosis of prediabetes in this study was primarily based on impaired fasting glucose, which may underestimate the true incidence of prediabetes compared to using additional diagnostic criteria like oral glucose tolerance test (OGTT) or glycated hemoglobin (HbA1c). Moreover, the average follow-up period of 3.12 years may not capture the long-term relationship between PP and prediabetes, and a longer follow-up duration would provide more robust results. Additionally, the data used in this study were derived from specific regions in China, which may limit the generalizability of the findings to the entire Chinese population. Future research should aim to include larger and more diverse populations to enhance the external validity of the results. Finally, apart from PP, our study has yet to incorporate more composite indicators such as hypertriglyceridemic waist-to-height ratio, TyG index, non-HDL cholesterol, residual cholesterol, among others. To enhance our understanding of diabetes, future research could further analyze the relationship between these composite indicators and both diabetes and prediabetes, thereby comprehensively advancing the prevention and treatment of diabetes. Thank you for your attention and support.

## Conclusion

We have made a novel discovery a non-linear relationship between PP and prediabetes risk. Specifically, we found that the risk of prediabetes significantly increases when PP ≥ 40 mmHg, while there is no significant change in prediabetes risk when PP < 40 mmHg. This finding emphasizes the importance of considering PP as a potential risk factor for prediabetes.

## Methods

### Data source

We retrieved raw data from the Dryad Digital Repository, a publicly accessible data repository. The dataset used in our study can be accessed at Dryad data repository (dataset: https://datadryad.org/stash/dataset/doi:10.5061%2Fdryad.ft8750v) and conducted a secondary analysis of a medical examination program using publicly available data provided by Chen et al.^27^.

### Study population

**Figure 4:**
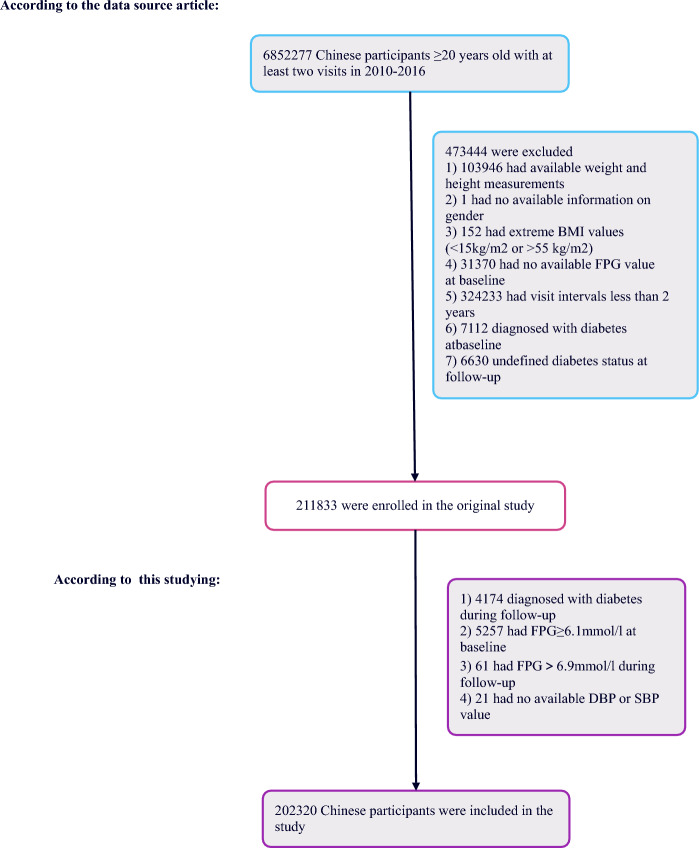
Flowchart of study participants.

The original dataset was derived from the Rich Healthcare Group’s computerized database in China, encompassing medical records from health check-ups conducted between 2010 and 2016 across 32 regions and 11 cities. Out of the initially enrolled 685,277 Chinese adults over 20 years old, each having at least two visits. Participants with follow-up fasting plasma glucose levels between 6.1 and 6.9 mmol/L and no new reports of diabetes were included in the study. Exclusions were made for individuals with a diabetes diagnosis at both baseline and follow-up, unclear diabetes status at follow-up, extreme BMI (outside the range of 15–55 kg/m^2^), missing baseline data for weight, height, sex, DBP, SBP, or fasting plasma glucose (FPG), or those with FPG levels over 5.6 mmol/L at baseline and exceeding 6.9 mmol/L during follow-up, including any new diabetes diagnoses. This resulted in a final cohort of 202,320 participants (Fig. 4).

### Ethics approval and consent to participate

The prior study received ethical approval from the Rich Healthcare Group Review Board, with informed consent waived for anonymous data. As a result, our secondary analysis, stemming from the original study, didn’t require additional ethical clearance. Our study adhered to all relevant guidelines and regulations.

### Variables

#### Pulse pressure (PP)

We considered several variables, with PP as the key variable of interest. PP was calculated as the difference between systolic blood pressure (SBP) and diastolic blood pressure (DBP)^28^.

#### Data collection

In the original study, participants’ blood pressure was measured using standard mercury sphygmomanometers in a resting state. Fasting venous blood samples were collected during each visit after at least 10 h of fasting. The Beckman 5800 automatic analyzer was utilized to measure various parameters, including plasma glucose, serum creatinine (Scr), aspartate aminotransferase (AST), triglycerides (TG), blood urea nitrogen (BUN), alanine aminotransferase (ALT), low-density lipoprotein cholesterol (LDL-C), and high-density lipoprotein cholesterol (HDL-C). The time taken for participants to return for one or more health check-ups, where they either returned to normal blood glucose levels or developed diabetes, was documented. Additionally, trained research personnel collected baseline data on alcohol consumption, smoking (1 for current, 2 for ever, 3 for never, and 4 for unclear), and family history of diabetes through standard questionnaires.

#### Outcome measures

Our primary outcome was the occurrence of prediabetes, defined by FPG levels in the range of 6.1–6.9 mmol/L at follow-up without reported incident diabetes^2^.

### Statistical analysis

We divided PP into four quartiles: Q1 ≤ 36 mmHg; 37 ≤ Q2 ≤ 42 mmHg; 43 ≤ Q3 ≤ 50 mmHg; Q4 ≥ 51 mmHg. And we employed ANOVA, Kruskal–Wallis tests, and chi-square tests to compare variables, including continuous and categorical data. The incidence rates were calculated using person-years, and the Kaplan–Meier method was used for survival and cumulative event rate analyses.

To assess the risk of prediabetes, we employed univariate and multivariate Cox regression analyses. Our models included crude, minimally-adjusted, and fully-adjusted models, with the final model excluding total cholesterol (TC) due to collinearity. Sensitivity analyses were conducted by excluding specific subgroups.

We also investigated the non-linear relationship between PP and prediabetes using smooth curve fitting and recursive algorithms to identify inflection points. Subgroup analyses were performed to assess interactions between different variables. All methods were carried out in accordance with relevant guidelines and regulations. For all analyses, Empower Stats was used, and a significance level of *P* < 0.05 was considered statistically significant.

## Data Availability

The raw data can be downloaded from the ‘DATADRYAD’ database (https://datadryad.org/stash/dataset/doi:10.5061%2Fdryad.ft8750v).

## References

[1] Echouffo-Tcheugui JB, Perreault L, Ji L (2023). Diagnosis and management of prediabetes: A review. JAMA.

[2] Rooney MR, Fang M, Ogurtsova K (2023). Global prevalence of prediabetes. Diabetes Care.

[3] Tabák AG, Herder C, Rathmann W (2012). Prediabetes: A high-risk state for diabetes development. Lancet.

[4] Mancia G, Fagard R, Narkiewicz K (2013). 2013 ESH/ESC guidelines for the management of arterial hypertension: The Task Force for the management of arterial hypertension of the European Society of Hypertension (ESH) and of the European Society of Cardiology (ESC). J. Hypertens..

[5] Whelton PK, Carey RM, Aronow WS (2018). 2017 ACC/AHA/AAPA/ABC/ACPM/AGS/APhA/ASH/ASPC/NMA/PCNA guideline for the prevention, detection, evaluation, and management of high blood pressure in adults: A report of the American College of Cardiology/American Heart Association Task Force on clinical practice guidelines. Hypertension.

[6] Meisinger C, Döring A, Heier M (2008). Blood pressure and risk of type 2 diabetes mellitus in men and women from the general population: The monitoring trends and determinants on cardiovascular diseases/cooperative health research in the region of Augsburg cohort study. J. Hypertens..

[7] Conen D, Ridker PM, Mora S (2007). Blood pressure and risk of developing type 2 diabetes mellitus: The women's health study. Eur. Heart J..

[8] Lee KS, Gi MY, Cha JA (2019). The relationship between pulse pressure, insulin resistance, and beta cell function in non-diabetic Korean adults. Prim. Care Diabetes.

[9] Xia Z, Song L, Fang D (2022). Higher systolic blood pressure is specifically associated with better islet beta-cell function in T2DM patients with high glycemic level. Cardiovasc. Diabetol..

[10] Anstadt GW (2020). Pulse pressure and isolated diastolic hypertension. JAMA.

[11] Vaccarino V, Holford TR, Krumholz HM (2000). Pulse pressure and risk for myocardial infarction and heart failure in the elderly. J. Am. Coll. Cardiol..

[12] Mitchell GF, Vasan RS, Keyes MJ (2007). Pulse pressure and risk of new-onset atrial fibrillation. JAMA.

[13] de Simone G, Roman MJ, Alderman MH (2005). Is high pulse pressure a marker of preclinical cardiovascular disease?. Hypertension.

[14] Baba Y, Ishikawa S, Kayaba K (2011). High pulse pressure is associated with increased risk of stroke in Japanese: The JMS Cohort Study. Blood Press..

[15] Safar ME, Totomoukouo JJ, Asmar RA, Laurent SM (1987). Increased pulse pressure in patients with arteriosclerosis obliterans of the lower limbs. Arteriosclerosis.

[16] Nargesi AA, Esteghamati S, Heidari B, Hafezi-Nejad N, Sheikhbahaei S, Pajouhi A, Nakhjavani M, Esteghamati A (2016). Nonlinear relation between pulse pressure and coronary heart disease in patients with type 2 diabetes or hypertension. J. Hypertens..

[17] Jia S, Wang X, Yao Q (2022). High pulse pressure is associated with an increased risk of diabetes in females but not in males: A retrospective cohort study. Biol. Sex Differ..

[18] Zhang L, Wang B, Wang C (2016). High pulse pressure is related to risk of type 2 diabetes mellitus in Chinese middle-aged females. Int. J. Cardiol..

[19] Beulens J, Rutters F, Rydén L (2019). Risk and management of pre-diabetes. Eur. J. Prev. Cardiol..

[20] Cederholm J, Wibell L (1992). Pulse pressure, mean blood pressure and impaired glucose tolerance—A study in middle-aged subjects. Ups. J. Med. Sci..

[21] Roengrit T, Sri-Amad R, Huipao N (2023). Impact of fasting blood glucose levels on blood pressure parameters among older adults with prediabetes. Sci. World J..

[22] Kawada T (2018). Risk factors for developing prediabetes. Diabetes Res. Clin. Pract..

[23] Gotoh M, Mizuno K, Ono Y (2005). High blood pressure, bone-mineral loss and insulin resistance in women. Hypertens. Res..

[24] Ferrannini E, Haffner SM, Stern MP (1991). High blood pressure and insulin resistance: Influence of ethnic background. Eur. J. Clin. Investig..

[25] Abramson JL, Weintraub WS, Vaccarino V (2002). Association between pulse pressure and C-reactive protein among apparently healthy US adults. Hypertension.

[26] Al-Shafei AI (2014). Ramadan fasting ameliorates arterial pulse pressure and lipid profile, and alleviates oxidative stress in hypertensive patients. Blood Press..

[27] Chen Y, Zhang XP, Yuan J (2018). Association of body mass index and age with incident diabetes in Chinese adults: A population-based cohort study. BMJ Open.

[28] Klassen PS, Lowrie EG, Reddan DN (2002). Association between pulse pressure and mortality in patients undergoing maintenance hemodialysis. JAMA.

